# Phloroglucinol Attenuates the Cognitive Deficits of the 5XFAD Mouse Model of Alzheimer’s Disease

**DOI:** 10.1371/journal.pone.0135686

**Published:** 2015-08-18

**Authors:** Eun-Jeong Yang, Sangzin Ahn, Junghwa Ryu, Moon-Seok Choi, Shinkyu Choi, Young Hae Chong, Jin-Won Hyun, Moon-Jeong Chang, Hye-Sun Kim

**Affiliations:** 1 Department of Pharmacology and Biomedical Sciences, College of Medicine, Seoul National University, Seoul, Republic of Korea; 2 Department of Microbiology, School of Medicine, Ewha Womans University, Seoul, Republic of Korea; 3 Department of Biochemistry, School of Medicine, Jeju National University, Jeju, Republic of Korea; 4 Department of Foods and Nutrition, College of Natural Science, Kookmin University, Seoul, Republic of Korea; 5 Seoul National University College of Medicine, Bundang Hospital, Bundang-Gu, Sungnam, Republic of Korea; 6 Neuroscience Research Institute, College of Medicine, Seoul National University, Seoul, Republic of Korea; Torrey Pines Institute for Molecular Studies, UNITED STATES

## Abstract

Alzheimer’s disease (AD) is the most common form of dementia among the elderly. Neuritic plaques whose primary component is amyloid beta peptide (Aβ) and neurofibrillary tangles which are composed of hyperphosphorylated tau, are known to be the neuropathological hallmarks of AD. In addition, impaired synaptic plasticity in neuronal networks is thought to be important mechanism underlying for the cognitive deficits observed in AD. Although various causative factors, including excitotoxicity, mitochondrial dysregulation and oxidative damage caused by Aβ, are involved in early onset of AD, fundamental therapeutics that can modify the progression of this disease are not currently available. In the present study, we investigated whether phloroglucinol (1, 3, 5—trihydroxybenzene), a component of phlorotannins, which are plentiful in *Ecklonia cava*, a marine brown alga species, displays therapeutic activities in AD. We found that phloroglucinol attenuates the increase in reactive oxygen species (ROS) accumulation induced by oligomeric Aβ_1–42_ (Aβ_1–42_) treatment in HT-22, hippocampal cell line. In addition, phloroglucinol was shown to ameliorate the reduction in dendritic spine density induced by Aβ_1–42_ treatment in rat primary hippocampal neuron cultures_._ We also found that the administration of phloroglucinol to the hippocampal region attenuated the impairments in cognitive dysfunction observed in 22-week-old 5XFAD (Tg6799) mice, which are used as an AD animal model. These results indicate that phloroglucinol displays therapeutic potential for AD by reducing the cellular ROS levels.

## Introduction

Alzheimer’s disease (AD) is the most common form of irreversible dementia, and can be neuropathologically identified by the hall mark lesions of AD; senile plaques consisting of extracellular deposits of Aβ and neurofibrillary tangles by accumulation of abnormal filaments of tau [1–6]. AD principally targets synapses, by which synaptic loss and dysfunction show strong connection to cognitive impairments in AD [7]. As the elderly population increases worldwide, the number of individuals with AD continues to increase http://onlinelibrary.wiley.com/doi/10.1111/bcp.12357/full-bcp12357-bib-0001. The World Health Organization has foreseen that 35.6 million people suffer from dementia and this number might be doubled by 2030. Though the accurate mechanisms underlying the impairments and/or loss of neurons in AD remain to be determined, emerging evidences have implied that elevation of reactive oxygen species (ROS) shown in the aged leads fairly to oxidative stress on microvascular system and neurons in central nerve system [8]. Oxidative stress results from an imbalance between antioxidant defenses and the intracellular accumulation of ROS, which may contribute to memory and cognitive function deficits in AD. Recent studies reported that this redox imbalance increases the production of amyloid beta peptide (Aβ) [9], thereby producing ROS, which act as a pro-oxidant to induce neuronal death in AD [10]. Furthermore, human AD brains have displayed an increase in the levels of oxidative stress markers and ROS production, negatively affecting synaptic plasticity [11–14]. Therefore, regulating oxidative damage may provide therapeutic efficacy in terms of synaptic plasticity and neuronal network function in AD.

Phloroglucinol is a polyphenol that is a component of phlorotannins which are sufficient in *E*. *cava* of the Laminariaceae family [15]. Several lines of evidence have demonstrated that the phloroglucinol exerts the positive effects against hydrogen peroxide-induced stress *in vitro* and *in vivo* [16–17]. Phlorogluicnol has been reported to reduce cell damage caused by hydrogen peroxide in lung fibroblast cells and gamma ray induced oxidative stress via an antioxidant mechanism [17]. Previously, we reported that phloroglucinol attenuates motor functional deficits in an animal model of Parkinson's disease by enhancing nuclear factor-like 2 (Nrf2) activity which regulates antioxidant proteins [18].

In the current study, we investigated the therapeutic effects of phloroglucinol on AD and demonstrated that phloroglucinol reduces oxidative stress induced by oligomeric Aβ_1–42_ (Aβ_1–42_) in the HT-22, hippocampal cell line_._ In addition, the reduction in dendritic spine density caused by either hydrogen peroxide or Aβ_1–42_ was significantly rescued by phloroglucinol in rat primary hippocampal neuron cultures. Furthermore, phloroglucinol attenuated memory deficits in the 5XFAD mouse model of AD based on the Morris water maze and T-maze tests.

As a whole, phloroglucinol display a therapeutic potential for AD patients as a ROS-scavenger.

## Materials and Methods

### Reagents

Phloroglucinol, hexafluoroisopropanol (HFIP) and Congo red reagent were purchased from Sigma Aldrich (St.Louis, MO, USA). A rabbit anti-Aβ monoclonal antibody was, from Cell Signaling Technology (Danvers, MA, USA). A mouse anti-synaptophysin monoclonal antibody was obtained from Merck Millipore (Darmstadt, Germany). A mouse monoclonal anti-PSD95 antibody was, from Thermo Scientific (Waltham, MA, USA). A rabbit polyclonal anti-MAP2 antibody was purchased from Santa Cruz Biotechnology (Dallas, TX, USA). A mouse anti-Aβ monoclonal antibody, 6E10, was purchased from Covance (Princeton, NJ, USA). A rat anti-neprilysin monoclonal antibody was purchased form R&D systems (Minneapolis, MN, USA). Aβ_1–42_ was obtained from American Peptide Company (Sunnyvale, CA, USA) and 3-(4,5-dimethylthiazol-2-yl)-2,5-diphenyltetrazolium bromide (MTT) and dimethyl sulfoxide (DMSO) were, from Duchefa Biochemie (B.V, Haarlem, Netherlands). Latate dehydrogenase (LDH) cytotoxicity assay kit was, from Thermo Scientific (Waltham, MA, USA). 7’-dichlorofluorescein diacetate (DCF-DA) was obtained from Invitrogen (Carlsbad, CA, USA). Dulbecco's modified Eagle's medium was purchased from Thermo scientific (Waltham, MA, USA). Fetal bovine serum (FBS), neurobasal medium, B27 supplement, GlutaMAX-I and penicillin/streptomycin were from Gibco (Carlsbad, CA, USA).

### Cell culture

Immortalized mouse hippocampal cell line, HT-22 cell line, which is a sub-line derived from parent HT4 cells that were originally immortalized from primary mouse hippocampal neuron culture (Lonza, Walkersville, MD) was grown in Dulbecco’s modified Eagle’s medium (DMEM), supplemented with 25 mM glucose, 2 mM L-glutamine, 2 mM pyruvate, penicillin (10 units/ml), streptomycin (10 mg/ml), and 10% (vol/vol) heat inactivated FBS in a humidified cell incubator (Binder, Germany) at 37°C under a 5% CO_2_ atmosphere. Primary hippocampal neuron cultures were prepared from embryonic day 18–19 (E18-19) pregnant Sprague-Dawley rats by dissociating with 0.25% trypsin and plated onto coverslips coated with poly-L-lysine. Neurons were grown in neurobasal medium supplemented with B27, 2mM GlutaMAX-I supplement and 100 μg/ml penicillin/streptomycin at 37°C in a humidified environment of 95% O_2_/5% CO_2_.

### Oligomeric Aβ preparation

We used a synthetic Aβ_1–42_ peptide that was determined to be >95% pure by RP-HPLC chromatography. Briefly, A_β1–42_ peptide was dissolved to 1 mmol/l in 100% HFIP, then HFIP was removed under vacuum, and the peptide was stored at −20°C. For oligomeric conditions, DMSO was added to bring the peptide to a final concentration of 100μmol/l, and the peptide was incubated at 4°C for 24 h.

### Cell viability measurements

Cell viability was determined by employing an MTT and LDH assays. MTT assay is based on the cleavage of a tetrazolium salt by mitochondrial dehydrogenase in viable cells. Cells were seeded in a 24-well plate at a concentration of 0.3×10^4^ cells/well, and Aβ_1–42_ (final concentration: 0.5–8 μM) or phloroglucinol (5–50 μg/ml) was added to the plate and incubated for an additional 24 h at 37°C to determine cytotoxicity. Subsequently, 20 μl of MTT stock solution (5 mg/ml) was added to each well for a total reaction volume of 200 μl and incubated for 3 h. The medium was removed and the formazan crystals in each well were dissolved in 400 μl DMSO. The absorbance was measured at 540 nm on a plate reader. The LDH assay was performed using an LDH assay kit (Thermo Scientific, Waltham, MA, USA). Briefly, 50 μl of medium was transferred to a 96 well plate and LDH activity was evaluated according to the manufacturer’s instructions.

### Detection of intracellular ROS

The cell permeable fluorescence dye, DCF-DA was employed to measure the level of intracellular ROS. Cells were seeded on a 24-well culture dish at 2.5×10^3^ cells/well. The cells were stimulated to 8 μM Aβ_1–42_ for 1 h, 3 h, 6 h or 9 h after the pretreatment of vehicle or 10 μg/ml phloroglucinol. Then, cell medium was removed and replaced with DMEM without phenol red containing 10 μM DCF-DA for 30 min in the dark at 37°C. The intensity of DCF-DA was detected by fluorescence spectroscopy (Olympus ix50, Hamburg, Germany) with excitation (495 nm) and emission (529 nm).

### Dendritic spine density analysis

Primary hippocampal neuron cultures were transfected with 3 μg CAG-IRES-mGFP in 18 mm Φ in 12-well plates. Primary hippocampal neuron cultures [*days per in vitro* 11 (DIV 11)] were transfected with CAG-IRES-mGFP. 250 nM Aβ_*1–42*_ for 72 h (DIV 15), 20 μM H_2_O_2_ for 1 h (DIV-17) and 10 μg/ml phloroglucinol for 24 h (DIV 17) were treated. The number of dendritic spines was evaluated at DIV 18. Fluorescent images were acquired by a confocal microscopy (LSM 510, Carl Zeiss, Jena, Germany) using identical settings for all samples. Spines were counted on 50 μm segments of secondary dendrites extending at least 50–100 μm beyond the cell body (soma).

### Animals

All of the experimental procedures were approved by the Animal Care Committee of Seoul National University (Approval number: SNUIBC-121018-1-1). Transgenic mice with 5XFAD mutations were purchased from Jackson Laboratories (strain: B6SJL-Tg [APPSwFlLon, PS1*M146L*L286V] 6799Vas/J) and maintained by crossing hemizygous transgenic mice with B6SJL F1 mice. Founder transgenic mice were identified by PCR, and non-transgenic littermates served as controls. 5XFAD mice express both mutant human APP695 which harbors the Swedish mutation (K670N, M671L), Florida mutation (I716V), and London mutation (V717I) and human PS1, which harbors two FAD mutations (M146L and L286V). Both transgenes are expressed under the control of the mouse Thy1 promoter to induce overexpression in the brain. These mice exhibit AD-related pathology earlier than other animal models, and amyloid deposition starts in the deep cortex and subiculum at 2 months of age. Synaptic marker proteins decrease at 4–9 months, and memory deficits are detected from 4–6 months of age [19–20]. Animal treatment and maintenance were performed in accordance with the Animal Care and Use Guidelines of Seoul National University, Seoul, Korea. Behavioral tests were performed in the Animal behavior lab of the Seoul National University.

### Stereotaxic surgery

Animals were anesthetized with isoflurane and placed on a Kopf stereotaxic frame (David Kopf Instruments, CA, USA). The injection of phloroglucinol (1.2 μmole) was bilaterally delivered through a 1.5 μl Hamilton syringe at the level of the dentate gyrus of the hippocampus, as following coordinates: anterior/posterior, −2.1 mm; medial/lateral, ± 0.15 mm; dorsal/ventral, −2.0 mm at a rate of 0.15 μl/min. The needle was kept in place for an additional 3 min before being slowly withdrawn. Administration route and dosage of phloroglucinol was determined based on a previous report [21].

### Morris water maze test

A circular water basin (120 cm in diameter) was filled with water (25 ± 2°C temperature) which was dyed with white milk, and a round platform (10 cm in diameter) was placed in the middle of one quadrant (the target quadrant). Considering the quadrant with the platform as North (N) and the other three quadrants West (W), South (S) and East (E), 4 visual cues were placed adjacent to the outer rim on the locations NW, NE, SW, SE. The animals were placed into each 3 quadrants (W, S, E) in a random order facing the wall, and was left to freely swim until they discovered the hidden platform. Mice are allowed a maximal time of 90 s to locate the platform. Finding the platform was defined as staying on it for at least 2 s. If the subject failed to find the platform, the mouse was guided and placed onto the platform for 10 s. Training to find the platform was performed for 4 consecutive days, and the whole sequence was videotaped with a digital camera attached to the ceiling. A probe test, which is a trial where the mice are placed in the basin that has the same visual cues but the platform is removed, was performed 3 days after the training was completed. The probe test was also videotaped and analyzed by Ethovision Software 8.5 (Noldus).

### Spontaneous alternation T-maze test

The dimension of each arm 30 x 15 x 7 cm and 7 x 7 cm center piece was constructed using acrylic plastic. After overnight food restriction, the trial was started by placing mice into the start arm facing the goal arms, and the mouse was left to freely explore until it was confined in an arm by placing an acrylic piece into the center after choosing the left or right goal arm. After 30 s, the center piece was removed and the mouse was moved back into the start arm, letting it choose again between the two open goal arms. The mice went through 2 trials per day with a 1 h interval for 2 consecutive days. Analysis was done by scoring, if the same goal arm was repeatedly chosen in the same trial score of 0, if different goal arms were chosen in the same trail score of 1 was given.

### Immunohistochemistry

Brains were cut into thicknesses of 30 μm. Brain sections were boiled in pH 6.0 citric acid for 10 min at 60°C and blocked with 10% normal goat serum in phosphate buffered saline (PBS). Brain sections were incubated with 6E10 antibody (1:500) diluted in blocking solution for overnight at 4°C. After incubation, sections were washed with PBS and incubated with secondary antibody diluted in PBS for 2 h at room temperature. The secondary antibody used was Alexa Fluor 488 goat anti-mouse IgG (1:200, Invitrogen, CA, USA). Next, samples were incubated with 4', 6-diamidino-2-phenylindole, (DAPI, 1:1000) for 30 min at room temperature. After three washes in PBS, sections were mounted on microscope slides in mounting medium. Confocal microscopic observation was performed using LSM 510 (Zeiss, Jena, Germany)

### Immunocytochemistry

Cells were fixed with 4% paraformaldehyde solution for 20 min at room temperature. After washing with PBS, the cells were permeabilized with PBS containing 0.1% Triton-X 100 and then blocked with blocking buffer (10% normal goat serum, and 0.1% Triton-X 100 in PBS) for 1 h at room temperature. The cells were then incubated with following primary antibodies, mouse anti-synaptophysin (1:200), mouse anti-PSD-95 (1:100), rabbit anti-MAP2 (1:100) for 4h at room temperature. After being washed with PBS, the cells were incubated with secondary antibodies for 2 h at room temperature. The secondary antibodies used were Alexa Fluor 488 goat anti-rabbit IgG (1:200, Invitrogen, CA, USA), Alexa Fluor 647 goat anti-mouse IgG (1:200, Invitrogen, CA, USA) and Alexa Fluor 555 goat anti-mouse IgM (1:200, Invitrogen, CA, USA). Then the cells were incubated in PBS containing 1 μM DAPI for 30 min at room temperature. The cells were then observed under a LSM 510 confocal microscope.

### Western Blotting

Primary hippocampal neuron cultures and brain tissues were lysed with RIPA buffer. 30 μg to 60 μg of protein was loaded on SDS-PAGE gel and transferred onto a nitrocellulose membrane. Then membranes were incubated in 5% skim milk for 1 h at room temperature. Next, the blots were incubated overnight at 4°C with primary antibodies, anti-Aβ (1:1000), anti-neprilysin (1:1000), anti-synaptophsyin (1:5000), anti-PSD-95 (1:1000) and anti-Glyceraldehyde 3-phosphate dehydrogenas (GAPDH) (1:2000) antibodies. The membranes were incubated with anti-mouse and anti-rabbit horseradish peroxidase-conjugated secondary antibody for 1 h at room temperature. The bands were detected by WestSave chemiluminescent detection kit (Young In Frontier, Seoul, Korea).

### Determination of internalization of Aβ _1–42_ by Congo red staining

Rat primary hippocampal neuron cultures were incubated with 250 nM Aβ_1–42_ for 48 h, followed by the addition of the vehicle or 10 μg/ml phloroglucinol for 24 h. The cells were fixed with a 4% paraformaldehyde solution for 20 min at room temperature. After being washed with PBS, the cells were stained with an alkaline solution of 0.1% filtered Congo red solution at room temperature for 5 min. Then the cells were washed with deionized water and mounted on microscope slides in mounting medium before being observed under a confocal microscope (LSM 510, Zeiss).

### Statistical analysis

All data are expressed as the means ± standard error of the mean (SEM) values. One-way ANOVA using Tukey post-hoc comparisons (IBM SPSS Statistics 20, IL) were used to determine statistical significance. The results were considered to be statistically significant if *p* < 0.05.

## Results

### Phloroglucinol reduces intracellular ROS accumulation induced by Aβ_1–42_ treatment

**Fig 1:**
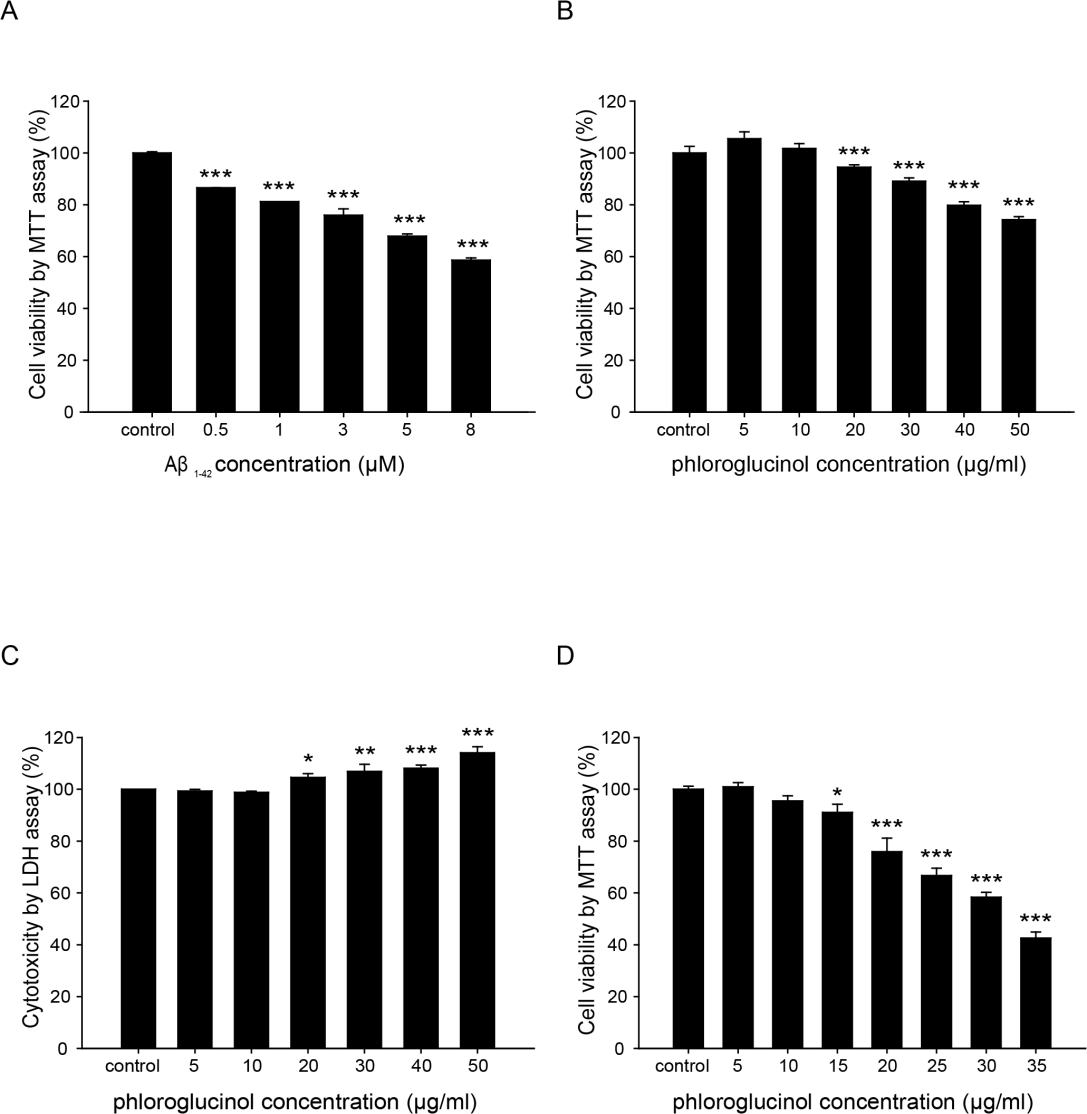
The effects of phloroglucinol and Aβ_1–42_ on cell viability were examined in HT-22 cells. (A) Cell viability was measured using MTT assays. HT-22 cells were treated with various Aβ_1–42_ concentrations (0, 0.5, 1, 3, 5 or 8 μM). Treatment with 8 μM Aβ_*1–42*_ was cytotoxic to approximately 50% of the cells. (B)(C) The HT-22 cells were treated with phloroglucinol at different concentrations (0, 5, 10, 20, 30, 40 or 50 μg/ml) for 24 h. Cell viability was evaluated using MTT (B) and LDH assays (C). (D) Primary cultured hippocampal neurons were treated with phloroglucinol at different concentrations (0, 5, 10, 15, 20, 25, 30 or 35 μg/ml) for 24 h. The percentage of cell viability was evaluated by an MTT assay. The statistical analyses were performed via one-way ANOVA, and the data are presented as the means ± SEM (*p<0.05, **p<0.01, ***p < 0.001).

The aggregation of oligomeric Aβ plays an important role in the pathophysiology of neurotoxicity and neurodegeneration in AD [22–25]. To measure the cytotoxic effects of Aβ_1–42_, we treated HT-22 hippocampal cells with Aβ_1–42_ at various concentrations (0.5 μM-8 μM) for 24 h and evaluated the cell viabilities by performing an MTT assay. Treatment with 8 μM Aβ_1–42_ was cytotoxic to approximately 50% of the cells (Fig 1A). Based on these results, we selected the concentration of 8 μM Aβ_*1–42*_ for the subsequent experiments on ROS.

Next, we evaluated whether phloroglucinol itself was toxic to the HT-22 cells and rat primary hippocampal neuron cultures by employing MTT and LDH assays. Of the doses used, ranging between 5 μg/ml and 50 μg/ml, treatment with 5 μg/ml or 10 μg/ml phloroglucinol for 24 h did not affect cell viability in the HT-22 cells and rat primary hippocampal neuron cultures. However, doses of phloroglucinol greater than 10 μg/ml reduced cell survival in a dose-dependent manner (Fig 1B, 1C and 1D). Based on these results, we selected 10 μg/ml phloroglucinol for the subsequent experiments (Fig 1B, 1C and 1D).

**Fig 2:**
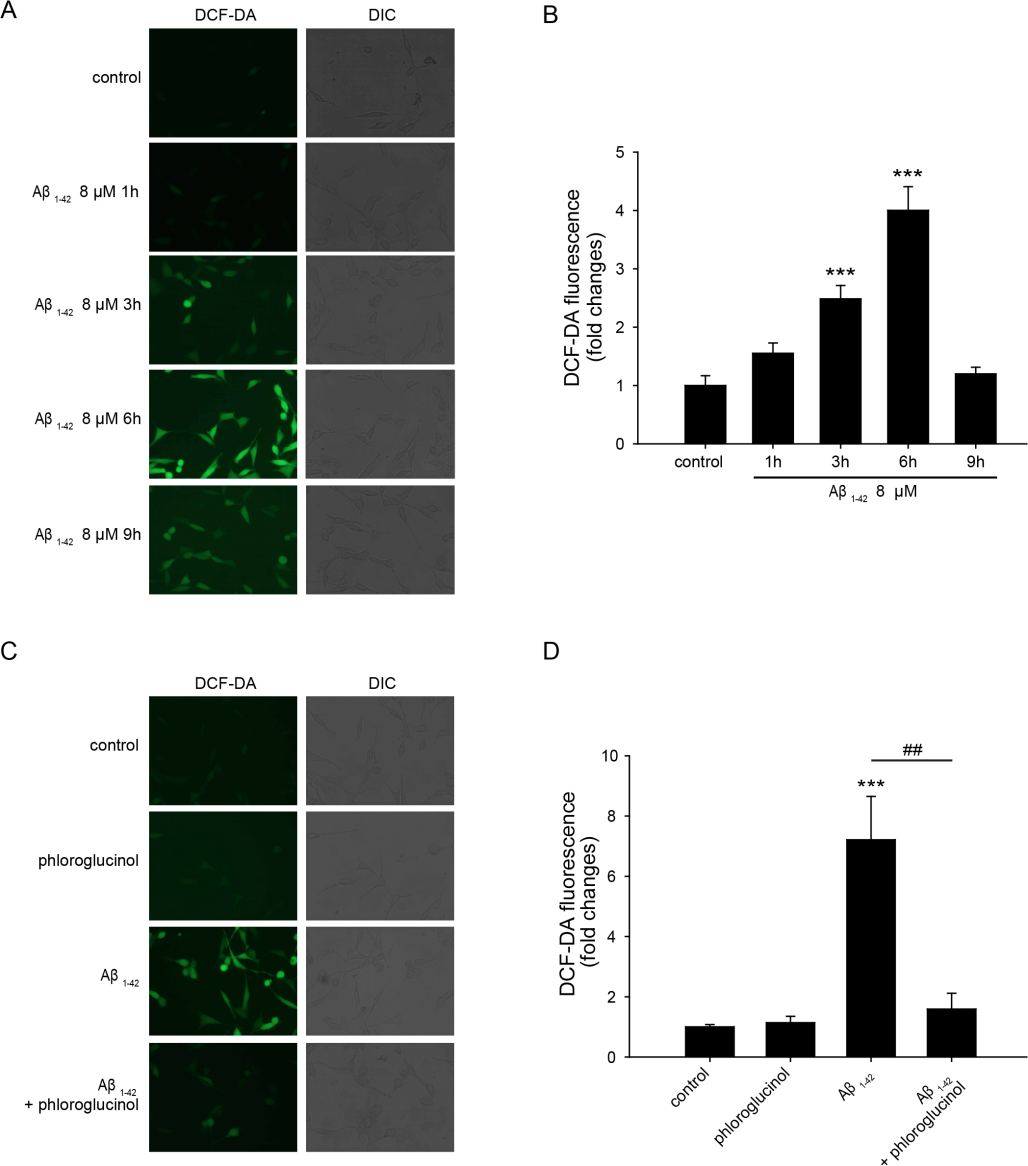
Phloroglucinol reduces intracellular ROS accumulation induced by Aβ_1–42_ treatment. (A)(C) The DCF-DA fluorescence in HT-22 cells were visualized via fluorescence microscopy. The fluorescent images were acquired with an Olympus ix50 microscope. (B)(D) Quantitation of DCF-DA fluorescence was performed. HT-22 cells were pretreated with 10 μg/mL phloroglucinol for 1 h, followed by the addition of 8 μM of Aβ_1–42_ to the cells. The DCF-DA fluorescence intensity in the cells pretreated with phloroglucinol was significantly lower (1.59 ± 0.52, n = 9, ^##^ *p*<0.01) compared with the Aβ_1–42_–treated cells (7.21 ± 1.44, n = 19). The statistical analyses were performed via one-way ANOVA, and the data are presented as the means ± SEM (***p < 0.001 vs. control, ^##^ *p*<0.01 vs. Aβ_1–42_-treated).

Next, we performed a DCF-DA assay to investigate the ROS levels after treatment with 8 μM Aβ_*1–42*_ for various periods (1 h, 3 h, 6 h, or 9 h) in the HT-22 cells. As shown in Fig 2A and 2B, a 4-fold (4.00 ± 0.40, n = 29, *p*<0.001) increase in the ROS level was observed after 6 h of Aβ_*1–42*_ treatment compared with the control (1.00 ± 0.17, n = 25). However, 9 h after treatment with Aβ_1–42_, the ROS levels (1.20 ± 0.11, n = 33) were reduced to the similar level of the control (Fig 2A and 2B). Subsequently, we examined the effects of phloroglucinol on oxidative stress induced by Aβ_1–42._ The cells were pretreated with 10 μg/ml phloroglucinol for 1 h and then treated with 8 μM Aβ_1–42_ for 6 h. The fluorescence intensity of DCF-DA was significantly enhanced approximately 7-fold (7.21 ± 1.44, n = 19, *p*<0.001) in the Aβ_1–42_–treated cells compared with the control (1.00 ± 0.08, n = 14). The DCF-DA fluorescence intensity in the cells pretreated with phloroglucinol was significantly lower (1.59 ± 0.52, n = 9, *p*<0.01) compared with the Aβ_1–42_-treated cells (7.21 ± 1.44, n = 19), indicating that phloroglucinol decreases the ROS accumulation induced by Aβ_1–42_ (Fig 2C and 2D). The discrepancy of the DCF-DA fluorescence intensity in the cells treated with Aβ_1–42_ in Fig 2B (approximately 4-fold increase) and Fig 2D (approximately 7-fold increase) may be because the DCF-DA fluorescence is affected by subtle differences in experimental conditions.

### Phloroglucinol rescues the reduced dendritic synaptic density induced by Aβ_1–42_ in primary hippocampal neuron cultures

Synaptic plasticity is a physiological process that underlies learning and memory at the cellular level [26]. One common measure of synaptic plasticity is dendritic spine density, which correlates with the strength of excitatory synapses [27]. The remodeling and maintenance of the synapse are based on the formation or elimination of dendritic spines in a neuronal activity-dependent manner [28]. Several studies have reported that ROS play a role in regulating synaptic plasticity and memory dysfunction [29–30].

**Fig 3:**
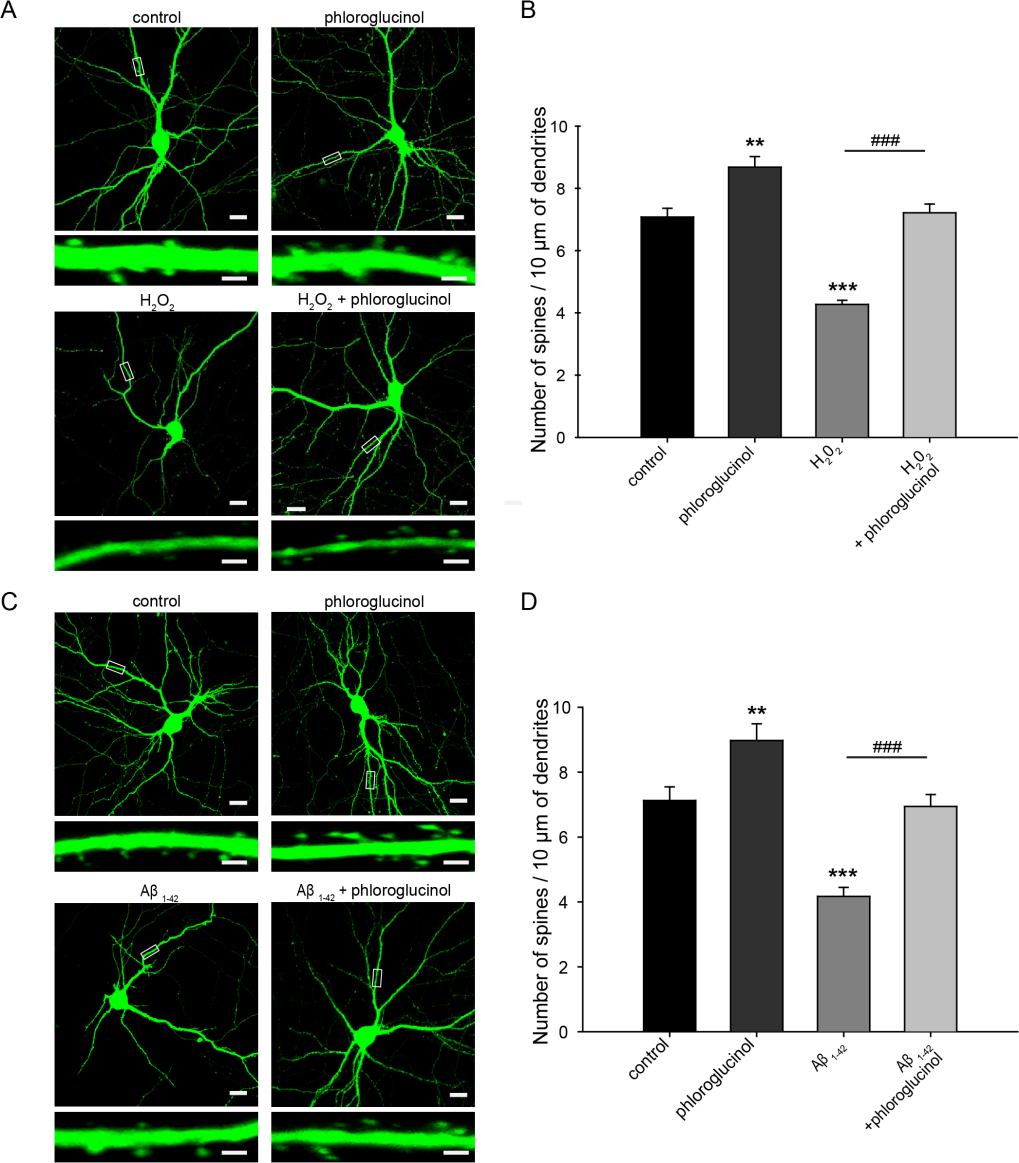
Phloroglucinol ameliorates the reduction in dendritic synaptic density- induced by Aβ_1–42_ in primary hippocampal neuron cultures. (A)(C) Representative images of dendritic spines on cultured primary hippocampal neurons at DIV 18. The dendritic segment, outlined with a white box (upper), is magnified to delineate spine morphology (bottom). The scale bars indicate 20 μm (low-scaled panel) and 1 μm (magnified panel). (B) Quantification of the spine density (secondary dendritic spines 50–100 μm from the soma) at DIV 18; the neurons were transfected at DIV 11 with IRES-mGFP. Treatment with hydrogen peroxide significantly decreased the spine density (4.27 ± 0.13 spines/10 μm, n = 43, ***p < 0.001) compared with the control treatment (7.07 ± 0.28 spines/10 μm, n = 50). The decrease in spine density induced by hydrogen peroxide was ameliorated by phloroglucinol (7.21 ± 0.28 spines/10 μm, n = 26, ^###^p<0.001 vs. hydrogen peroxide-treated). (D) The dendritic spine density of neurons treated with Aβ_1–42_ was significantly reduced (4.17 ± 0.28 spines/10 μm, n = 31, ***p < 0.001) compared to the control neurons (7.12 ± 0.28 spines/10 μm, n = 31). Adding phloroglucinol to the primary hippocampal neurons treated with Aβ_1–42_ significantly alleviated the reduction in dendritic spine density (6.93 ± 0.36 spines/10 μm, n = 41, ^###^p<0.001 vs. Aβ_1–42_-treated). The statistical analyses were performed via one-way ANOVA, and the data are presented as the means ± SEM (**p < 0.01 or ***p < 0.001 vs. control, ^###^p<0.001 vs. hydrogen peroxide or Aβ_1–42_-treated).

First, we examined whether dendritic spine density is affected by oxidative stress by treating primary hippocampal neuron cultures with hydrogen peroxide. The exposure of primary hippocampal neurons to 20 μM hydrogen peroxide for 1 h reduced dendritic spine density (4.27 ± 0.13 spines/10 μm, n = 43, *p*<0.001) compared with the control (7.07 ± 0.28 spines/10 μm, n = 50). Treatment with 10 μg/ml phloroglucinol rescued the reduced spine density induced by 20 μM hydrogen peroxide treatment (7.21 ± 0.28 spines/10 μm, n = 26, *p*<0.001) (Fig 3A and 3B).

Dendritic spine and synaptic loss are well documented in AD. Aβ decreases dendritic spine density in primary neurons [31]. In addition, a decrease in dendritic spine density was observed in the brains of AD animal models such as 5XFAD mice [32]. We confirmed that treatment with 250 nM Aβ_1–42_ for 72 h induced a significant reduction in dendritic spine density at DIV 18 in rat primary hippocampal neuron cultures (4.17 ± 0.28 spines/10 μm, n = 31, *p*<0.001) compared with the control (7.13 ± 0.42 spines/10 μm, n = 31). Treatment of phloroglucinol ameliorated the reduced dendritic spine density induced by 250 nM Aβ_1–42_ (6.93 ± 0.36 spines/10 μm, n = 41, *p*<0.001) (Fig 3C and 3D). In these experiments, treatment with phloroglucinol alone appeared to significantly increase the spine density (8.40 ± 0.37 spines/10 μm, n = 54 and 8.77 ± 0.45 spines/10 μm, n = 36, *p*<0.01) (Fig 3B and 3D, respectively), compared to the controls, likely because phloroglucinol attenuates oxidative stress exerted in normal culture conditions. Based on this result, we deduced that the dendritic spine density was reduced due to oxidative stress in rat primary hippocampal neuron cultures and that this reduction was alleviated by adding phloroglucinol, which attenuated oxidative stress.

**Fig 4:**
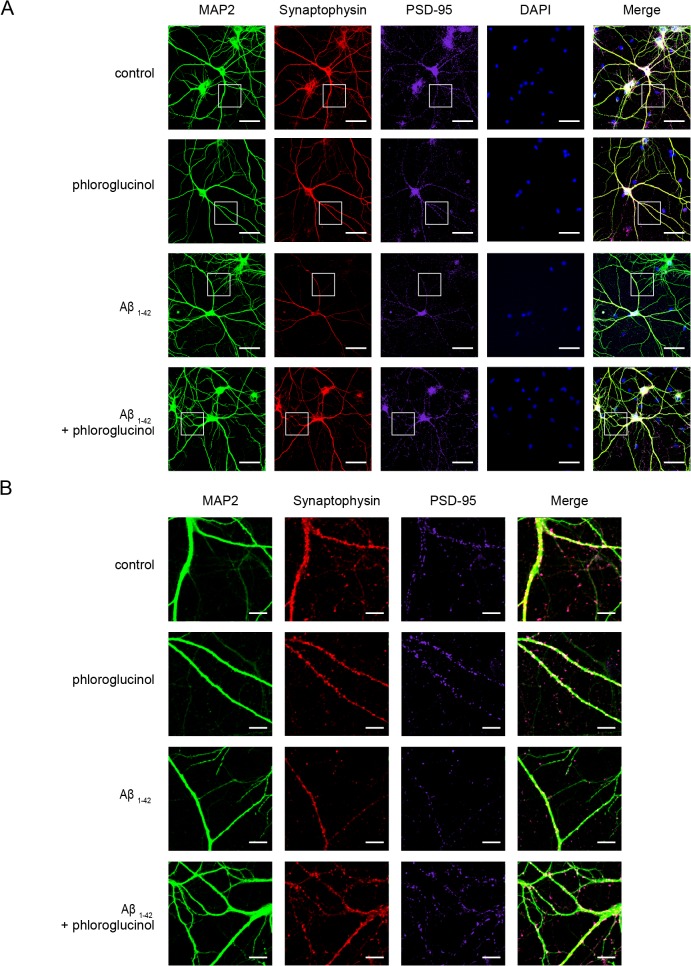
Phloroglucinol ameliorates the reduction in the synaptophysin and PSD-95 immunoreactivities induced by Aβ_1–42_ in primary hippocampal neuron cultures. (A) Synaptophysin, PSD-95 and MAP2 immunoreactivities were visualized with immunocytochemistry at DIV 18 in rat primary hippocampal neuron cultures treated with vehicle, phloroglucinol and Aβ_1–42_ with or without phloroglucinol treatment. (B) The dendritic segment, outlined with a white box (A), is magnified to delineate spine morphology (B). The scale bars indicate 50 μm (A) and 10 μm (B). Phloroglucinol ameliorated the reduced immuroreactivity observed for synaptophysin and PSD-95 in the rat primary hippocampal neuron cultures treated with Aβ_1–42_. Representative images of 7 independent experiments were shown.

**Fig 5:**
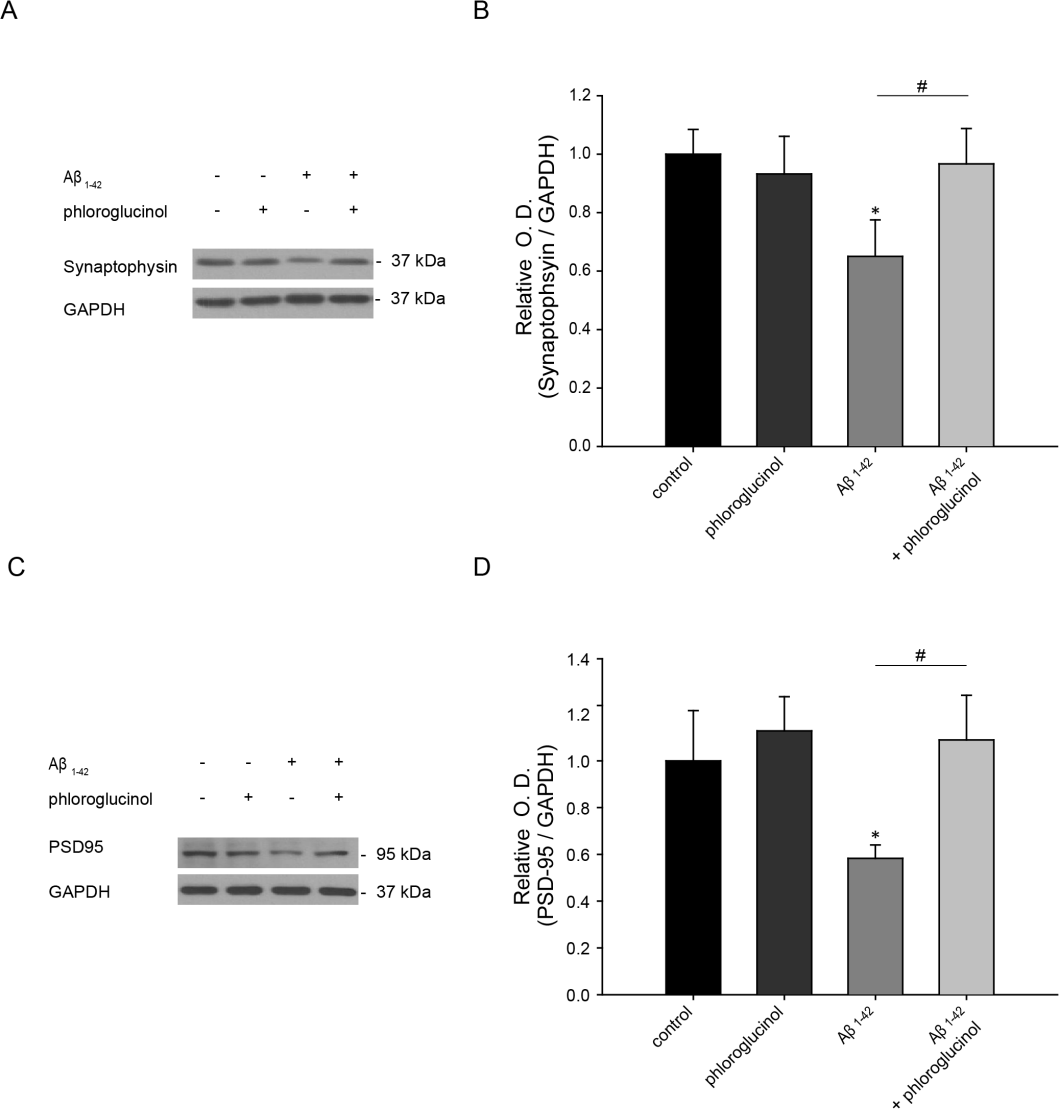
Phloroglucinol ameliorates the reduction in synaptophysin and PSD protein induced by Aβ_1–42_ in primary hippocampal neuron cultures. (A) (C) Synaptophysin and PSD-95 protein levels were measured by Western blotting in the primary hippocampal neurons treated with vehicle, phloroglucinol and Aβ_1–42_ with or without phloroglucinol treatment. (B) (D) A quantitative graph was shown for relative optical density for synaptophysin (B) and PSD-95 (D) vs. GAPDH. Aβ_1–42_ treatment reduced the protein level of synaptophysin (0.65 ± 0.12 fold, n = 6) compared to the controls (1 ± 0.08, n = 8 *p<0.05). The level of synaptophysin, reduced by Aβ_1–42_ was significantly restored by phloroglucinol treatment (0.96 ± 0.12, n = 9 *p<0.05). Similarly, the reduced level of PSD-95 protein caused by Aβ_1–42_ (0.58 ± 0.05, n = 8) was significantly restored by phloroglucinol treatment (1.08 ± 0.15, n = 8 ^#^ *p*<0.05). The statistical analyses were performed via one-way ANOVA, and the data are presented as the means ± SEM (*p < 0.05 vs. control, ^#^p<0.05 vs. Aβ_1–42_-treated).

In addition to the quantitation of dendritic spine density, we evaluated the effects of phloroglucinol on a pre-synaptic marker, synaptophysin, and a post-synaptic marker, PSD-95 by immunocytochemistry and Western blotting at DIV 18. We stained dendrites with anti-MAP2 antibody, and also immunostained with anti-synaptophysin antibody for presynaptic terminals and with anti-PSD-95 for postsynaptic density. As shown in Fig 4A and 4B, phloroglucinol ameliorated the reduced immuroreactivity observed for synaptophysin and PSD-95 in the rat primary hippocampal neuron cultures treated with Aβ_1–42_. We confirmed this result by Western blotting. Aβ_1–42_ treatment reduced the protein level of synaptophysin (0.65 ± 0.12 fold, n = 6, *p*<0.05) compared to the control (1 ± 0.08, n = 8). The level of synaptophysin, reduced by Aβ_1–42_ was significantly restored by phloroglucinol treatment (0.96 ± 0.12, n = 9, *p*<0.05), compared by Aβ_1–42_ alone treatment (Fig 5A and 5B). Similarly, the reduced level of PSD-95 protein caused by Aβ_1–42_ (0.58 ± 0.05, n = 8, *p*<0.05) was significantly restored by phloroglucinol treatment (1.08 ± 0.15, n = 8) (Fig 5C and 5D).

### Phloroglucinol does not affect the internalization of Aβ _1–42_ in rat primary hippocampal neuron cultures

In the early phases of AD (i.e., mild cognitive impairment and prodromal AD), intraneuronal accumulation of Aβ is found in the brain regions critically involved in the cognitive deficits [33–34]. Moreover, intraneuronal Aβ accumulation reduces synaptic protein expression [35] and glutamatergic synaptic function at both the presynaptic and the postsynaptic levels [36], indicating that Aβ internalization from the extracellular space and its intracellular accumulation play a pivotal role in synaptic dysfunction. To understand the effect of phloroglucinol on the internalization of Aβ in rat primary cultured hippocampal neuron cultures, the neurons were exposed to 250 nM Aβ_1–42_ for 48 h and treated with phloroglucinol for another 24 h. Afterwards, the neurons were stained with Congo red for 5 min. As shown in S1A Fig, the neuron treated with Aβ_1–42_ alone showed a marked internalization of the peptide, however, the treatment with phloroglucinol did not affect the internalization of the Aβ_1–42_ peptide. We confirmed this result by Western blotting (S1B and S1C Fig).

### Phloroglucinol attenuates the impairments in memory deficits in the 5XFAD mouse model of AD

**Fig 6:**
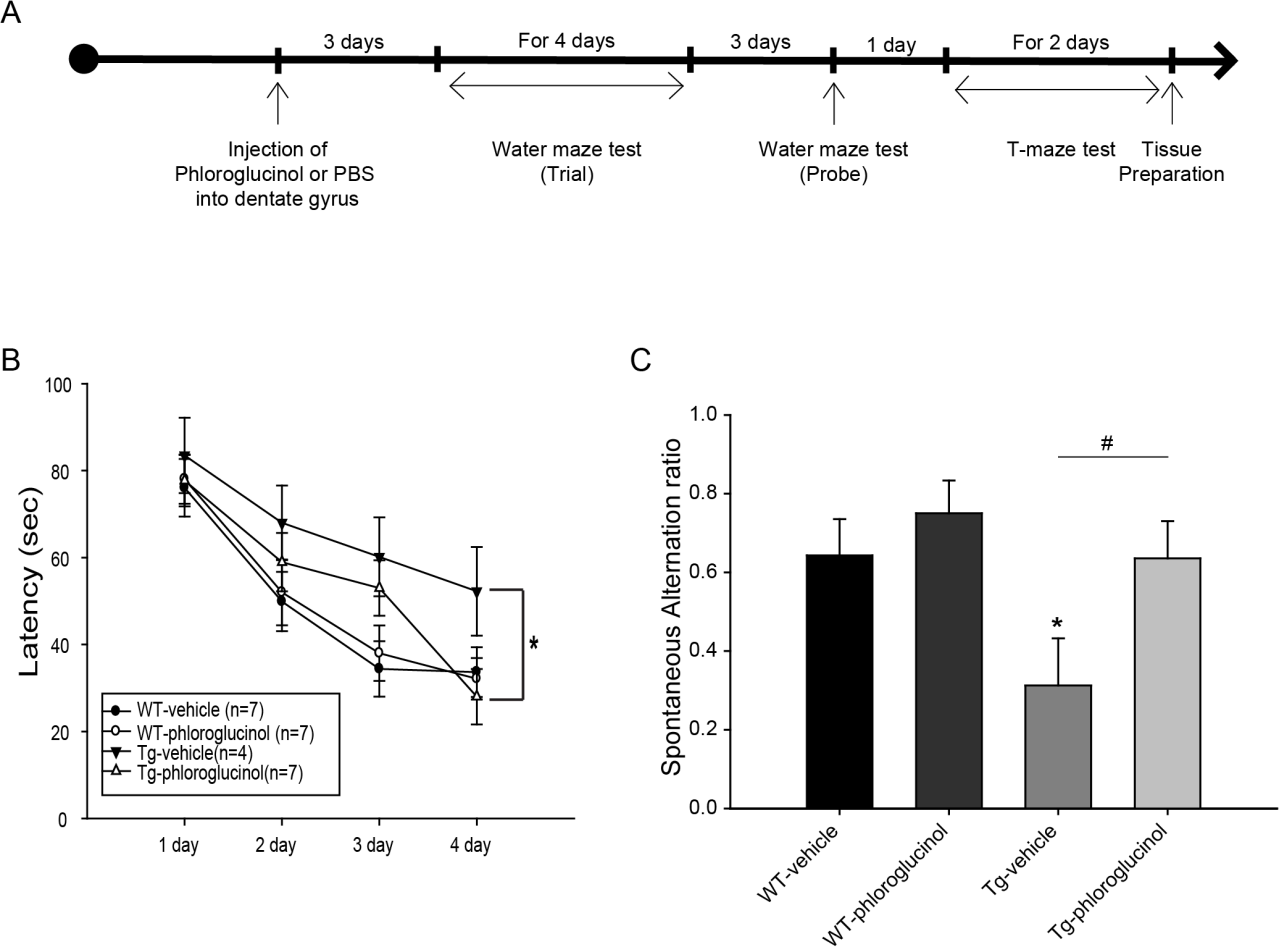
Phloroglucinol rescues cognitive dysfunction and working memory in the 5XFAD Tg mouse model. (A) A schematic of the experimental scheme is shown. (B) The latency for each mouse to reach the hidden platform was recorded. Compared with the WT (33.67 ± 5.73 s, n = 7), the 5XFAD mice exhibited a longer latency (52.25 ± 10.21 s, n = 4, *p < 0.05) to locate the hidden platform. Injection of phloroglucinol into the 5XFAD mice resulted in an improved performance on the 4^th^ day of the training stage (28.00 ± 6.36 s, n = 7); this result is not significantly different from that of the WT mice (33.66 ± 5.73 s, n = 7). (C) The 5XFAD mice exhibited significantly reduced levels of spontaneous alternation performance on the T-maze (0.31 ± 0.11, n = 4, *p < 0.05), compared to the WT mice (0.64 ± 0.09, n = 7). Injection of phloroglucinol ameliorated this reduction in the spontaneous alternation performance shown by the 22-week-old 5XFAD mice (0.71 ± 0.09, n = 7, ^#^ *p* < 0.05). The statistical analyses were performed via one-way ANOVA, and the data are presented as the means ± SEM (*p < 0.05 vs. WT-vehicle, ^#^p<0.05 vs. Tg-vehicle).

Next, we investigated the effects of phloroglucinol *in vivo* in an AD animal model. We injected 1.2 μmole phloroglucinol, dissolved in 1.5 μl PBS into the dentate gyrus using a stereotaxic apparatus. As shown in the schematic of the experimental scheme of Fig 6A, 3 days after the injection, we performed a Morris water maze test on the wild type (WT)-vehicle, WT-phloroglucinol, 5XFAD (Tg)–vehicle and Tg-phloroglucinol groups to examine their spatial learning and memory capacities. Compared with the WT (33.67 ± 5.73 s, n = 7), the Tg mice exhibited a longer latency (52.25 ± 10.21 s, n = 4, *p* < 0.05) to locate the hidden platform. The Tg mice that were given phloroglucinol exhibited a significantly shorter escape latency (28 ± 0.36 s, *p*<0.05) than Tg mice given the vehicle (52.25± 10.21 s) on day 4 of the learning stage (Fig 6B). 3 days later, we performed a probe test in which the platform was removed. No significant differences in the time spent in the quadrant that had contained the platform was detected between the groups of mice (data not shown). 11 days after the injection (Fig 6C), the T-maze test, which is a hippocampus-dependent memory task, was performed to evaluate whether phloroglucinol affects working memory in the AD Tg mouse model. We investigated the spontaneous alternation ratio, which represents spatial working memory. The results showed a decreased alternation ratio in the Tg mice (0.31 ± 0.12, n = 4, *p*<0.05) compared with WT (0.64 ± 0.09, n = 7). This reduction (0.31 ± 0.12, n = 7, *p*<0.05) observed in the Tg mice was significantly restored by the administration of phloroglucinol (0.71 ± 0.09).

To investigate whether the phloroglucinol-mediated improvement in cognitive function impairments was due to changes in the Aβ level, we assayed the level of Aβ by immunohistochemistry and Western blotting in hippocampi from WT and Tg mice treated with the vehicle or with phloroglucinol. No significant differences in Aβ level were observed between the animal groups (S2A, S2B and S2C Fig). We also checked the level of neprilysin, a major Aβ degrading enzyme, by Western blotting, but no significant differences were observed between the animal groups (S2B Fig), indicating that phloroglucinol did not affect Aβ generation and/or degradation.

### The reduced protein level of synaptophysin and PSD-95 was restored by phloroglucinol in 5XFAD Tg mouse model

**Fig 7:**
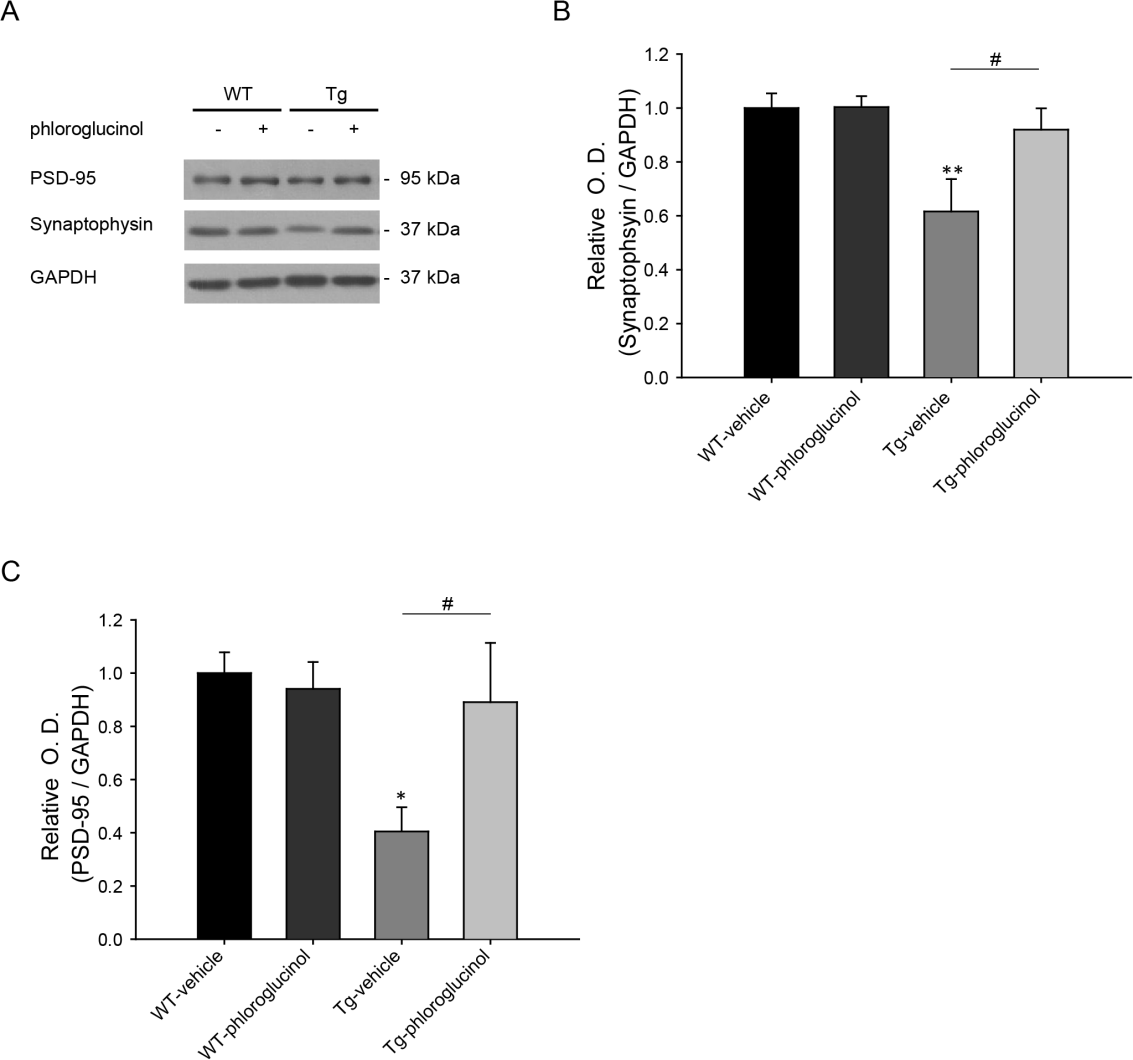
The reduced protein level of synaptophysin and PSD-95 was restored by phloroglucinol in 5XFAD Tg mouse model. (A) Synaptophysin protein levels were evaluated by Western blotting of the hippocampal tissue lysates from WT and 5XFAD mice treated with the vehicle or phloroglucinol. (B) PSD-95 protein levels were evaluated with Western blotting in the hippocampus from WT and 5XFAD mice treated with the vehicle or phloroglucinol. Tg-phloroglucinol mice showed rescued synaptophysin (0.91 ± 0.08, ^#^ *p*<0.05, n = 9, vs.Tg-vehicle) and PSD-95 (0.89 ± 0.22, n = 8, ^#^ *p*<0.05 vs. Tg-vehicle) protein levels, compared with the Tg-vehicle mice. The statistical analyses were performed via one-way ANOVA, and the data are presented as the means ± SEM (*p < 0.05, **p < 0.01 vs. WT-vehicle, ^#^p<0.05 vs. Tg- vehicle).

Next, we investigated the levels of synaptophysin and PSD-95 protein in the hippocampal tissues of the four animal groups. As shown Fig 7, Tg-vehicle mice showed decreased levels of both synaptophysin (0.61 ± 0.12, n = 5, *p*<0.01, Fig 7A and 7B) compared to the WT-vehicle (1 ± 0.054, n = 9) and PSD-95 (0.40 ± 0.09, n = 6, p<0.05, Fig 7B and 7C) compared with WT-vehicle (1 ± 0.078, n = 9). However, Tg-phloroglucinol mice showed rescued synaptophysin (0.91 ± 0.08, n = 9, *p*<0.05) and PSD-95 (0.89 ± 0.22, n = 8, *p*<0.05) protein levels, compared with the Tg-vehicle mice.

## Discussion

Oxidative stress is an important causative factor in the pathogenesis of neurodegenerative diseases, including AD [12]. Several studies have implicated oxidative stress in Aβ-induced neurotoxicity [37–43]. Aβ increases the levels of hydrogen peroxide and lipid peroxides, both *in vitro* and *in vivo* [9], suggesting that Aβ acts as a potent inducer of oxidative stress, resulting in synaptic impairment and neuronal loss. Increased oxidative stress caused by Aβ may contribute to the decline in cognitive performance in AD.

In this study, we investigated the therapeutic potential of phloroglucinol, a component of the edible brown algae *E*. *cava*, for AD using *in vitro* and *in vivo* models. Phloroglucinol is a monomeric compound in phlorotannins which are extracted from *Ecklonia species* [44]. Several studies have reported that phloroglucinol directly inhibits ROS generation [45] and exerts anti-oxidative effects by upregulating the levels of antioxidant enzymes such as superoxide dismutase and glutathione peroxidase [46]. Previously, we reported that phloroglucinol protects against hydrogen peroxide- or gamma ray- induced oxidative damages in *in vitro and/or in vivo* [16, 17, 47, 48]. Moreover, our group unveiled that phloroglucinol attenuates the motor functional deficits of a 6-hydroxydopamine-induced Parkinson’s disease animal model through Nrf-2 activation [18].

Our report is the first study to investigate the effects of phloroglucinol on the Aβ-induced oxidative stress and dendritic spine density reduction in neurons followed by cognitive function deficits in an AD animal model. First, we examined the effects of phloroglucinol on ROS accumulation induced by Aβ_1–42_ in HT-22 cells, and we found that phloroglucinol reduces intracellular ROS levels in these cells treated with 8 μM Aβ_*1–42*_ for 6 h, based on the DCF-DA assay (Fig 2C and 2D).

Based on these results and those of previous studies [17, 18], we hypothesize that phloroglucinol’s antioxidant efficacy may derive from three rationales, as follows. First, phloroglucinol includes a polyphenolic structure with an electron-rich composite that is prone to transit into electron-donation reactions with oxidizing agents to formulate intermediate phenoxyl radical (PhO·) species. Those radicals are sequentially stabilized through delocalization of the unpaired electron to the *ortho* and *para* sites of the benzene ring. Moreover, phenoxyl radicals can be stabilized through next two processes; hydrogen bonding with an adjacent hydroxyl group and dimerization (phenol coupling) to generate new CC or CO linkages [15]. The antioxidant activity of phloroglucinol might derive from the intrinsic stability of phenolic structures. Second, phloroglucinol may enhance the levels of hydrogen peroxide degrading enzymes, such as catalase and glutathione, by increasing the transcriptional activity of Nrf2. Third, phloroglucinol itself can upregulate the activities of antioxidant enzymes, as shown in our previous report [18].

A recent report indicates that Aβ-induced ROS accumulation causes synaptic impairments, including reduced dendritic spine density, by inactivating the protein kinase A (PKA)/CREB signal transduction pathway [49]. Dendritic spines serve as the fundamental basic units that comprise neuronal networks in the brain [50]. Here, we investigated the effects of treatment with hydrogen peroxide or Aβ on dendritic spine density in rat primary hippocampal neuron cultures. Treatment with either 20 μM hydrogen peroxide for 1 h or 250 nM Aβ_*1–42*_ for 72 h decreased the dendritic spine density in primary hippocampal neuron cultures. We found that phloroglucinol ameliorated the reduction in dendritic spine density induced by hydrogen peroxide or Aβ_1–42_ in these neuronal cultures (Fig 3).

Next, we evaluated the effects of phloroglucinol on the cognitive deficits of the 5XFAD AD animal model. At 3 days and 11 days after injection of phloroglucinol into the hippocampus, the Morris water maze and T-maze tests were performed on the 22-week-old 5XFAD mice. Phloroglucinol remarkably shortened the escape latency times on the 4^th^ training day (28 ± 0.36 s) compared to the vehicle (52.25 ± 10.21 s) on the Morris water maze test. In addition, the animals’ impaired reference and working memory, as evaluated by the T-maze test, were relieved by phloroglucinol administration to the 5XFAD mice (Fig 6C). In addition, the Tg-phloroglucinol mice showed rescued levels of synaptophysin and PSD-95, compared to the Tg-vehicle mice (Fig 7). Meanwhile, no significant difference in Aβ and neprilysin levels were observed between the animal groups administered the vehicle or phloroglucinol (S2 Fig).

In this study, we administered phloroglucinol directly into the dentate gyrus region of the hippocampus, which has been implicated as the sub-region most sensitive to the effects of advancing age [51] and also known as one of the regions where neuritic plaques are frequently seen, together with stratum radiatum of the hippocampus and fimbra [52]. In the near future, we will investigate the pharmacokinetics of phloroglucinol, and, based on those results, we will confirm the effects of phloroglucinol after administrating systemically via ip injection or oral application.

Due to the extended life span of the global population, more and more individuals are suffering from AD. Nevertheless, the need for available therapeutics or appropriate treatment strategies is not currently met. Much effort has been focused on the development of therapeutics to potentially ameliorate impairments in cognitive dysfunction in AD.

Our data showed that phloroglucinol displays therapeutic potential in AD and may delay the onset or inhibit the progression of AD by attenuating the deficits in cognitive function by acting as an anti-oxidant. Based on our results, we speculate that the beneficial effects of phloroglucinol on AD behavioral phenotype is not due to a direct effect on generation and/or degradation of Aβ, but due to protective effects towards the reduction in dendritic spine density and synaptic proteins such as synaptophysin and PSD-95.

Therefore, treatment with antioxidants may represent an approach that targets several different molecular events implicated in the pathogenesis of AD.

## Supporting Information

S1 Fig Phloroglucinol does not affect internalization of Aβ_1–42_ in rat primary hippocampal neuron cultures. (A)Rat primary hippocampal neuron cultures were incubated with 250 nM Aβ_1–42_ for 48 h, followed by the addition of the vehicle or 10 μg/m phloroglucinol for 24 h. After being washed with PBS, the cells were stained with Congo red for 5 min. The primary neuron cultures treated with Aβ_1–42_ alone showed much more marked internalization of the peptide, however, treatment with phloroglucinol did not affect the internalization of the Aβ_1–42_ peptide. Scale bar indicates 50 μm. (B) Aβ levels in the rat primary hippocampal neuron cultures treated with vehicle, phloroglucinol and Aβ_1–42_ with or without phloroglucinol were measured by Western blotting. (C) A quantitative graph of Aβ protein level was shown.
(TIF)

S2 Fig Phloroglucinol does not alter Aβ level in 5XFAD mice. (A) Aβ plaques were detected with 6E10 antibody in the hippocampal regions of the WT-vehicle, WT-phloroglucinol, Tg-vehicle and Tg-phloroglucinol mice groups. Arrows indicate neuritic plaques stained with 6E10 antibody. Representative images of 3 independent experiments are shown. Scale bar indicates 100 μm. (B) Aβ and neprilysin protein levels were measured by Western blotting of the hippocampal tissue lysates of WT and Tg mice injected with vehicle or phloroglucinol. (C) A quantitative graph of Aβ protein level was shown. WT-vehicle n = 7, WT-phloroglucinol n = 7, Tg-vehicle n = 4, Tg-phloroglucinol n = 7). The data are presented as the means ± SEM.
(TIF)
