# Supplementary figures and images for: Phloroglucinol Attenuates the Cognitive Deficits of the 5XFAD Mouse Model of Alzheimer’s Disease
Source: PLoS One. 2015 Aug 18;10(8):e0135686. doi: 10.1371/journal.pone.0135686 (PMC4540482; doi:10.1371/journal.pone.0135686)

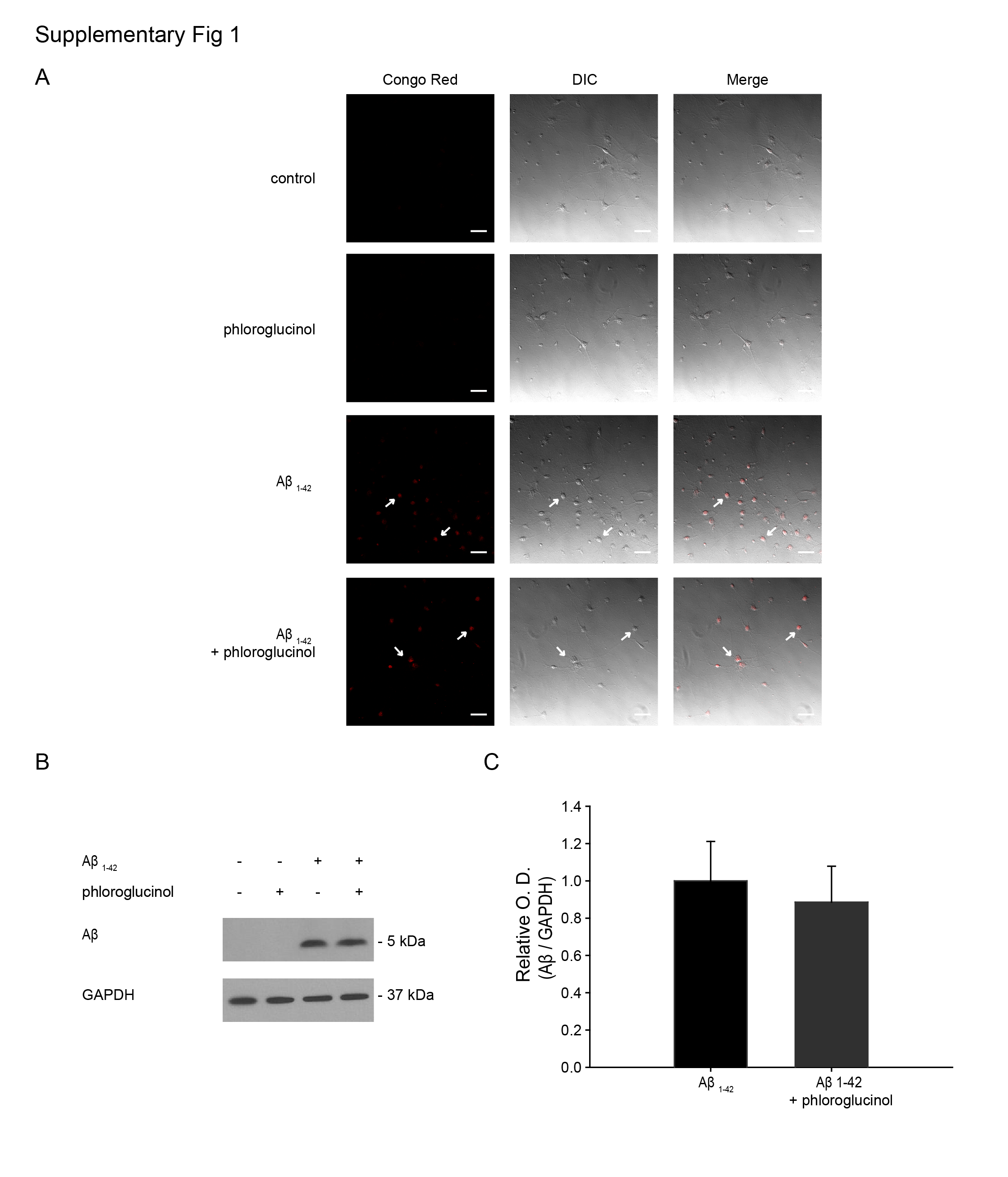

Supplement: S1 Fig — (A)Rat primary hippocampal neuron cultures were incubated with 250 nM Aβ1–42 for 48 h, followed by the addition of the vehicle or 10 μg/m phloroglucinol for 24 h. After being washed with PBS, the cells were stained with Congo red for 5 min. The primary neuron cultures treated with Aβ1–42 alone showed much more marked internalization of the peptide, however, treatment with phloroglucinol did not affect the internalization of the Aβ1–42 peptide. Scale bar indicates 50 μm. (B) Aβ levels in the rat primary hippocampal neuron cultures treated with vehicle, phloroglucinol and Aβ1–42 with or without phloroglucinol were measured by Western blotting. (C) A quantitative graph of Aβ protein level was shown. (TIF) [file pone.0135686.s001.tif]

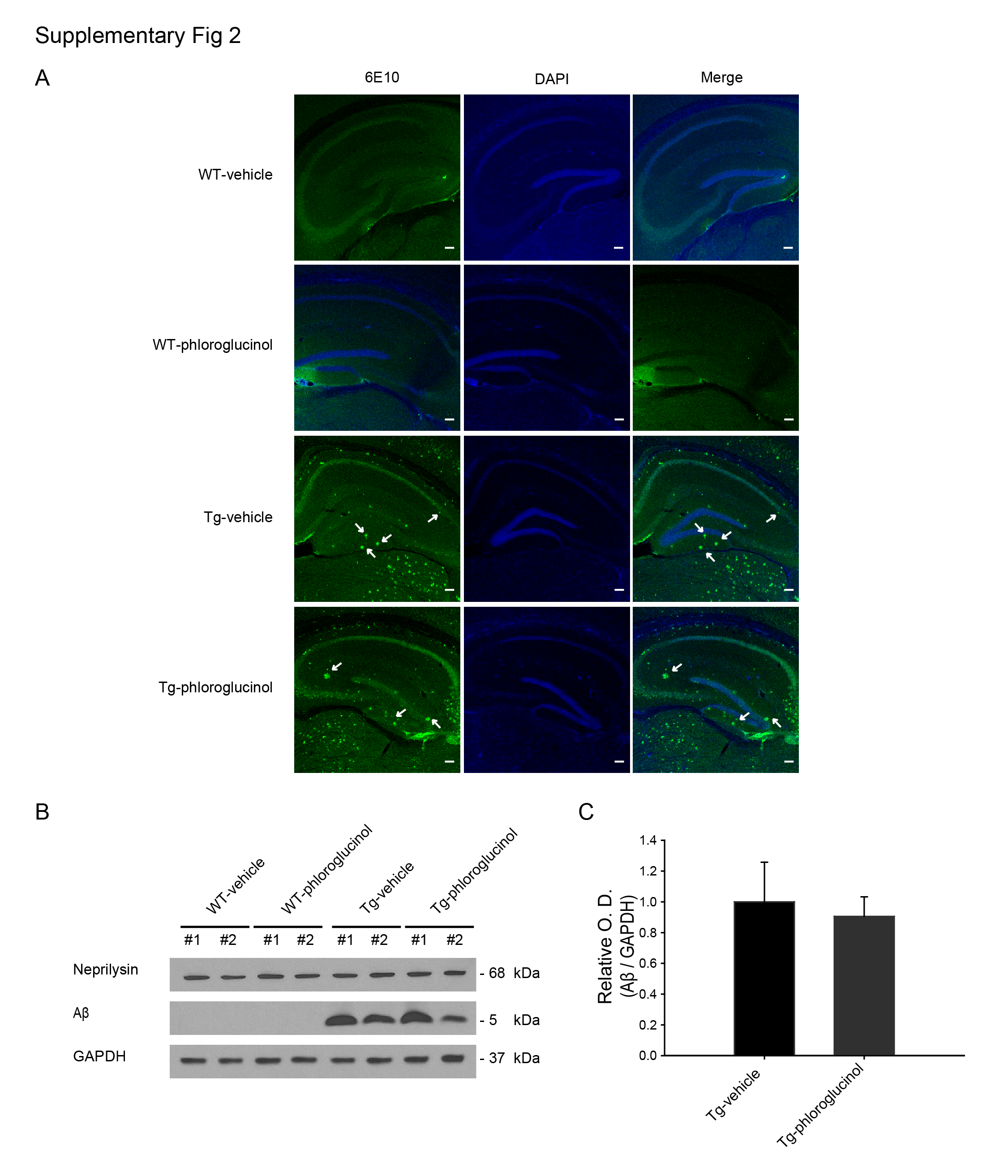

Supplement: S2 Fig — (A) Aβ plaques were detected with 6E10 antibody in the hippocampal regions of the WT-vehicle, WT-phloroglucinol, Tg-vehicle and Tg-phloroglucinol mice groups. Arrows indicate neuritic plaques stained with 6E10 antibody. Representative images of 3 independent experiments are shown. Scale bar indicates 100 μm. (B) Aβ and neprilysin protein levels were measured by Western blotting of the hippocampal tissue lysates of WT and Tg mice injected with vehicle or phloroglucinol. (C) A quantitative graph of Aβ protein level was shown. WT-vehicle n = 7, WT-phloroglucinol n = 7, Tg-vehicle n = 4, Tg-phloroglucinol n = 7). The data are presented as the means ± SEM. (TIF) [file pone.0135686.s002.tif]
